# TAp63γ and ΔNp63γ are regulated by RBM38 via mRNA stability and have an opposing function in growth suppression

**DOI:** 10.18632/oncotarget.18463

**Published:** 2017-06-13

**Authors:** Wensheng Yan, Yanhong Zhang, Xinbin Chen

**Affiliations:** ^1^ The Comparative Oncology Laboratory, Schools of Veterinary Medicine and Medicine, University of California at Davis, Davis, California, USA

**Keywords:** p63, p63γ, RBM38, RNA binding protein, mRNA stability

## Abstract

The *p63* gene is expressed as TAp63 from the P1 promoter and as ΔNp63 from the P2 promoter. Through alternative splicing, five TA and five ΔN isoforms (α-ε) are expressed. Isoforms α-β and δ share an identical 3’ untranslated region (3’UTR) whereas isoform γ has a unique 3’UTR. Recently, we found that RBM38 RNA-binding protein is a target of p63 and RBM38 in turn regulates p63α/β expression via mRNA stability. However, it is uncertain whether p63γ has a unique biological activity and whether p63γ is regulated by RBM38. Here, we found that the levels of ΔNp63γ transcript and protein are induced upon overexpression of RBM38 but decreased by RBM38 knockdown. Conversely, we found that the levels of ΔNp63β transcript and protein are decreased by ectopic expression of RBM38 but increased by RBM38 knockdown, consistent with our previous report. Interestingly, RBM38 increases the half-life of p63γ mRNA by binding to a GU-rich element in p63γ 3’UTR. In contrast, our previous studies showed that RBM38 decreases the half-life of p63α/β mRNAs by binding to AU-/U-rich elements in their 3’UTR. We also found that knockout of p63γ in ME180 and HaCaT cells, in which ΔNp63 isoforms are predominant, inhibits cell proliferation and migration, suggesting that ΔNp63γ has a pro-growth activity. In contrast, we found that knockout of TAp63γ in MIA PaCa-2 cells, in which TAp63 isoforms are predominant, promotes cell proliferation, migration, and inhibits cellular senescence. Taken together, we conclude that ΔNp63γ has an oncogenic potential whereas TAp63γ is a tumor suppressor.

## INTRODUCTION

The *p63* gene, a member of the p53 family transcription factors, is expressed as TA and ΔN isoforms. The expression of TAp63 is under control of the P1 promoter located immediately upstream of the first exon whereas the expression of ΔNp63 is under control of the P2 promoter in intron 3 [1]. TAp63 contains an N-terminal activation domain, which is homologous to the N-terminal activation domain in p53 [2]. Thus, TAp63 has an activity similar to p53, such as in inducing p21 [3]. ΔNp63 lacks the activation domain conserved in p53 but carries a unique activation domain in the N-terminus, which is composed of 14 unique N-terminal residues and the proline-rich domain [4]. Thus, ΔNp63 possesses not only an activity distinct from TAp63 but also a dominant negative activity towards p53, TAp63 and TAp73, suggesting a role for ΔNp63 isoforms as oncogenes [2, 5, 6]. Through C-terminal alternative splicing, five TA and five ΔN isoforms (α-ε) are expressed [1]. Isoforms α, β, and δ share an identical 3’ untranslated region (3’UTR) from exon 15, whereas isoforms γ and ε have their unique 3’UTRs from exons 11 and 10a, respectively [1].

p63 has been shown to possess dual roles in tumorigenesis due to the presence of TA and ΔN isoforms in cells. Many studies have highlighted the oncogenic properties of ΔNp63. ΔNp63 is frequently found to be amplified and overexpressed in squamous cell carcinomas [7, 8]. ΔNp63α overexpression promotes cell proliferation *in vitro* and tumor growth *in vivo* [9, 10]. In addition, ΔNp63α is found to exhibit a survival function in squamous cell carcinoma by repressing TAp73-dependent pro-apoptotic activity [5]. The role of ΔNp63 in promoting tumorigenesis may be partially due to its transcriptional activity [11, 12]. Previously, we found that GPX2 and BMP7, two direct targets of ΔNp63, inhibit oxidative stress-induced apoptosis in a p53-dependent manner and are required for survival of tumor cells [13, 14]. On the other hand, a study showed that *p63*^*+/–*^ mice are predisposed to develop spontaneous tumors [15], suggesting that the *p63* gene acts as a tumor suppressor. Consistently, TAp63 is found to induce senescence and suppress tumorigenesis in the model of *TAp63* conditional knockout mice [16]. In addition, downregulation of TAp63 enhances epithelial-to-mesenchymal transition [17], a process which plays pivotal roles in promoting cancer invasion and metastasis [18]. Since most studies were focused on the function of p63α, the role for p63γ in tumorigenesis and metastasis is still uncertain and thus will be investigated in this study.

Increased or decreased expression of p63 is associated with the function of TA or Δp63 in tumorigesesis [7-10, 16, 17]. Thus, the regulation of p63 expression at the levels of transcription and posttranscription may affect the functions of p63. The regulation of mRNA stability by RNA-binding proteins is one of the major mechanisms to posttranscriptionally control gene expression. For example, RBM38, a RNA-binding protein and a target of the p53 family, is able to stabilize p21 transcript and consequently suppresses cell growth [19-22]. Previously, we also showed that RBM38 is capable of posttranscriptionally regulating the expression of the p53 family members, including p53, p63, and p73 [23-25]. Specially, we found that ectopic expression of RBM38 inhibits, whereas knockdown of RBM38 stabilizes, mRNA stability of p63α and β isoforms through binding to the AU/U-rich elements in their 3’UTRs [24]. Since p63γ has a unique 3’UTR and RNA binding proteins mainly regulate the mRNA stability or translation via binding to responsive elements in 3’UTR [19, 24-26], we explored whether RBM38 regulates the mRNA stability of p63γ isoform.

## RESULTS

### p63γ expression is differentially regulated by RBM38

**Figure 1:**
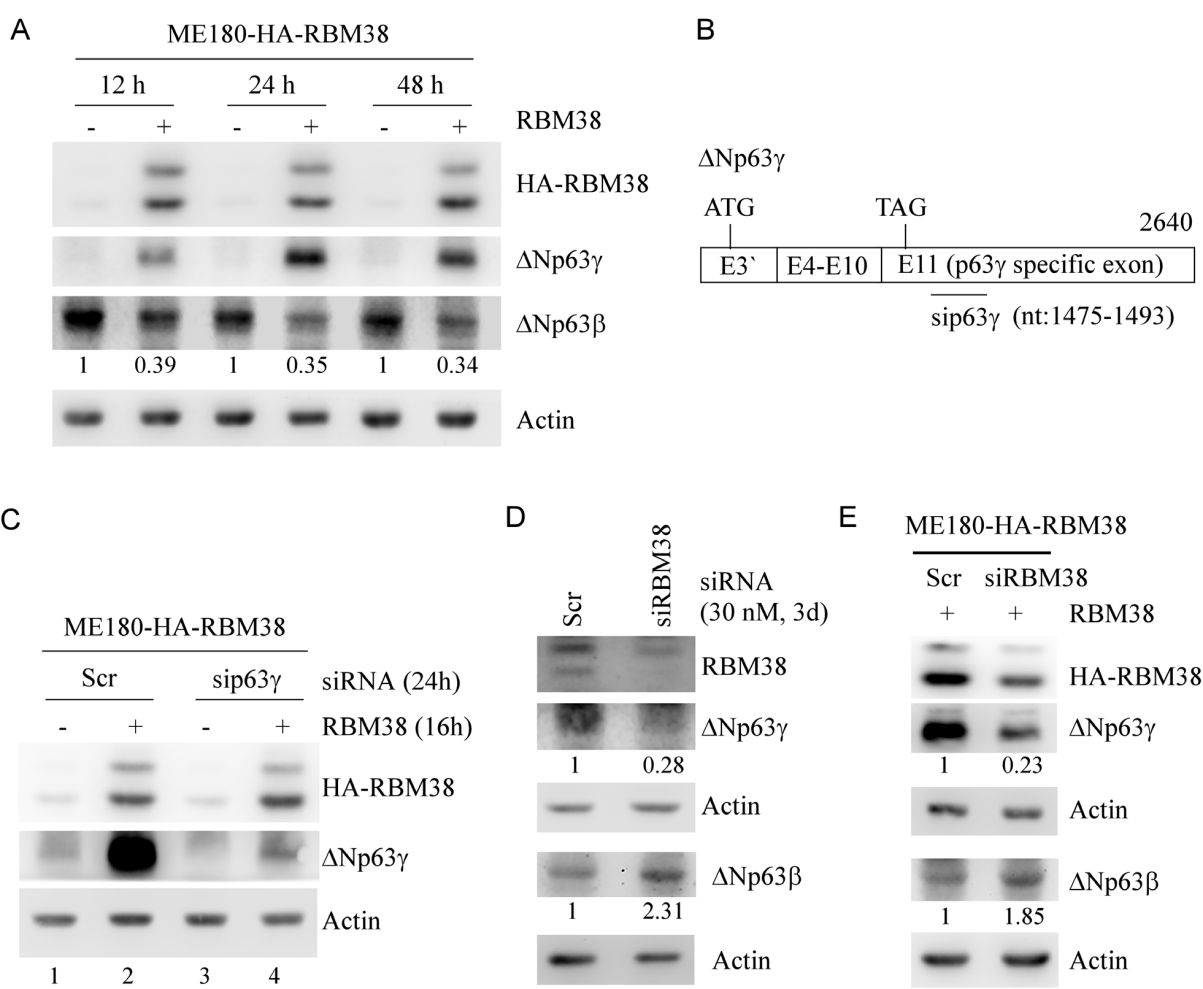
The levels of ΔNp63γ and ΔNp63β proteins are differentially regulated by RBM38 **A.** Ectopic expression of RBM38 increases the level of ΔNp63γ protein but decreases the level of ΔNp63β protein. Western blots were prepared with extracts from ME180-HA-RBM38 cells, which were uninduced (-) or induced (+) to express RBM38 for 12, 24, or 48 h, and then probed with antibodies against p63, HA tag, and actin, respectively. The basal level of ΔNp63β in cells without RBM38 induction was arbitrarily set at 1.0 and the fold change was shown below each lane. **B.** Schematic presentation of ΔNp63γ transcript and the location of siRNA against ΔNp63γ. **C.** Specific p63γ siRNA decreases the expression of basal and RBM38-induced ΔNp63γ protein. Western blots were prepared with extracts from ME180-HA-RBM38 cells, which were transfected with scrambled siRNA or siRNA again p63γ for 8 h and then uninduced (-) or induced (+) to express RBM38 for 16 h, and then probed as in *A*. **D.** Knockdown of endogenous RBM38 decreases ΔNp63γ expression but increased ΔNp63β expression. Western blots were prepared with extracts from ME180 cells, which were transfected with scrambled siRNA or siRNA again RBM38 for 3 d, and then probed with antibodies against RBM38, p63, and actin, respectively. The level of protein was quantified as in *A*. **E.** Knockdown of exogenous RBM38 inhibits RBM38-induced ΔNp63γ expression with increased expression of ΔNp63β. Western blots were prepared with extracts from ME180-HA-RBM38 cells, which were transfected with scrambled siRNA or siRNA again RBM38 for 3 d, induced to express RBM38 for 12 h, and then probed as in *A*. The level of protein was quantified as in *A*.

Recently, we found that RBM38 regulates p63α/β expression via mRNA stability through the binding of RBM38 to the AU-/U-rich elements in p63α/β 3’UTRs [24]. Since p63γ isoform contains a unique 3’UTR from exon 11, we wanted to determine whether RBM38 also regulates p63γ. To test this, ME180 and HaCaT cell lines, which can inducibly express HA-tagged RBM38 under the control of a tetracycline-regulated promoter as described previously [24], were used. We would like to note that ΔNp63 isoforms are predominantly expressed in ME180 and HaCaT cells [24, 27]. We found that in ME180 cells, the level of ΔNp63γ protein was increased at 12, 24, and 48 h after ectopic expression of RBM38 (Figure 1A). Conversely, the level of ΔNp63β protein was decreased by ectopic expression of RBM38 (Figure 1A), consistent with our previous report [24]. Likewise, ectopic expression of RBM38 increased the level of ΔNp63γ protein, but decreased the level of ΔNp63β protein, in HaCaT cells (Supplementary Figure 1A). To confirm that the RBM38-induced protein band is ΔNp63γ instead of other isoforms of p63, a scrambled siRNA or an siRNA specifically targeting the 1475- to 1493-nt region in the unique exon 11 of ΔNp63γ (Figure 1B) was transfected into ME180 and HaCaT cells with/without inducible expression of RBM38. We found that the levels of the basal and RBM38-induced ΔNp63γ protein were decreased by p63γ siRNA in ME180 cells (Figure 1C. compare lanes 1 and 2 with 3 and 4, respectively) and HaCaT cells (Supplementary Figure 1B, compare lanes 1 and 2 with 3 and 4, respectively).

To examine whether endogenous RBM38 has an effect on ΔNp63γ expression, the level of ΔNp63γ protein was measured in ME180 cells with RBM38 knockdown. We showed that compared to the scrambled siRNA, RBM38 siRNA decreased the level of endogenous RBM38 (Figure 1D). Importantly, RBM38 knockdown resulted in a marked reduction of ΔNp63γ protein along with increased expression of ΔNp63β protein (Figure 1D). Similarly, knockdown of tetracycline-induced RBM38 also significantly decreased the level of ΔNp63γ protein but increased the level of ΔNp63β protein (Figure 1E). These data indicate that p63α/β and p63γ are differentially regulated by RBM38.

### RBM38 increases the expression of p63γ *via* enhancing the stability of p63γ mRNA

**Figure 2:**
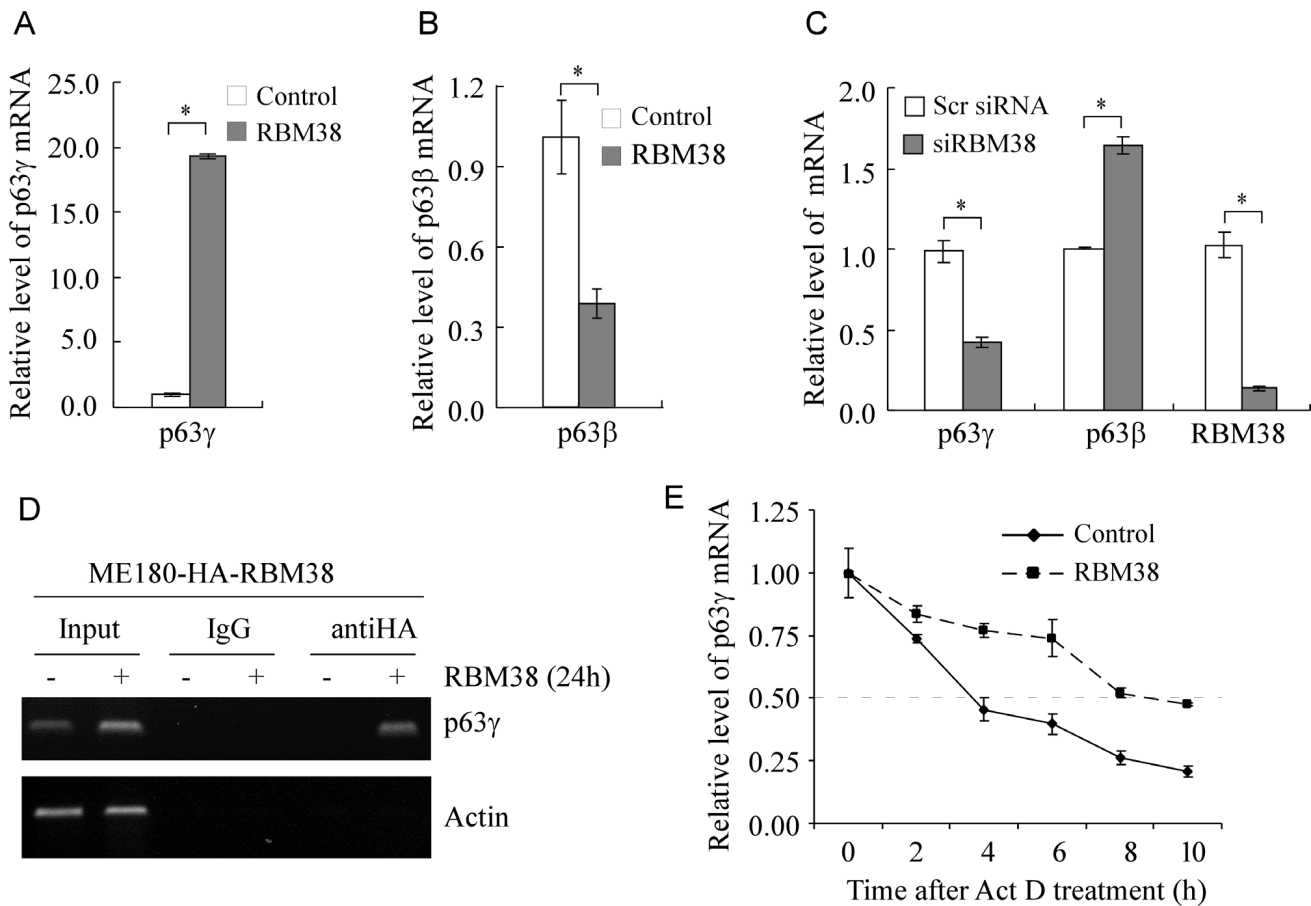
RBM38 regulates p63γ expression *via* mRNA stability **A.** The level of p63γ transcripts is increased by ectopic expression of RBM38. Quantitative RT-PCR (qRT-PCR) was performed with total RNAs isolated from ME180-HA-RBM38 cells, which were uninduced (-) or induced (+) to express RBM38 for 24 h. The relative level of p63γ mRNA was normalized by level of actin mRNA from three independent experiments. Asterisk indicates a significant difference (*p* = 1.2E-08). **B.** The level of p63β transcripts is decreased by ectopic expression of RBM38. qRT-PCR was performed as in *A*. Asterisk indicates a significant difference (*p* = 0.00172). **C.** Knockdown of RBM38 decreases the level of p63γ transcripts but increases the level of p63β transcripts. qRT-PCR was performed as in *A* except that total RNA was isolated from ME180 cells, which were transfected with scrambled siRNA or siRNA again RBM38 for 3 d. Asterisk indicates a significant difference (p63γ, *p* = 0.000185; p63β, *p* = 0.00389; RBM38, *p* = 5.0E-05). **D.** p63γ mRNA is recognized by RBM38. RNA immunoprecipitation assay was performed with ME180-HA-RBM38 cells uninduced (-) or induced (+) to express HA-tagged RBM38 for 24 h. Anti-HA was used to precipitate HA-tagged RBM38-RNA complexes along with IgG as a control. The binding of RBM38 to p63γ transcripts was measured by RT-PCR. Actin was used as a negative control. **E.** The half-life of p63γ transcript is prolonged by ectopic expression of RBM38. ME180-HA-RBM38 cells were uninduced (-) or induced (+) to express RBM38 for 12 h followed by treatment with 5 μg/ml of actinomycin D (Act D) for 0, 2, 4, 6, 8, or 10 h. The relative half-life of p63γ transcript was calculated from triplicate samples and presented as Mean ± S.D.

As a RNA-binding protein, RBM38 may directly bind to and stabilize ΔNp63γ mRNA and consequently increase its expression. Thus, both quantitative and regular RT-PCRs were performed and showed that the level of p63γ transcripts was markedly increased by ectopic expression of RBM38 in ME180 (Figure 2A and Supplementary Figure 2C) and HaCaT cells (Supplementary Figure 2, A and C). In contrast, the level of p63β transcripts was significantly decreased by ectopic expression of RBM38 in ME180 (Figure 2*B* and Supplementary Figure 2C) and HaCaT cells (Supplementary Figure 2, B and C), consistent with previous report [24]. To examine whether endogenous RBM38 has an effect on the expression of p63γ mRNA, the level of p63γ transcripts was measured by quantitative RT-PCR in ME180 cells with RBM38 knockdown. We showed that compared to scrambled siRNA, the level of endogenous RBM38 transcripts was decreased by RBM38 siRNA (Figure 2C). Importantly, RBM38 knockdown resulted in a marked reduction of p63γ transcripts along with a significant increase of p63β transcripts (Figure 2C).

To explore the mechanism by which RBM38 increases the level of p63γ transcripts, we sought to determine whether RBM38 associates with p63γ transcripts in vivo by RNA immunoprecipitation assay. We showed that p63γ transcripts were detected in anti-RBM38 but not in control IgG immunoprecipitates (Figure 2D). Next, we explored whether RBM38 has an effect on p63γ mRNA stability. To test this, ME180 cells were uninduced or induced to express RBM38 and then treated with actinomycin D to inhibit nascent RNA synthesis. Quantitative RT-PCR analysis showed that the relative half-life of p63γ mRNA was increased from around 3.7 to 8.6 h (Figure 2E). This result suggests that RBM38 increases p63γ expression by prolonging the half-life of p63γ mRNA.

### Identification of RBM38 responsive element in p63γ 3’UTR

**Figure 3:**
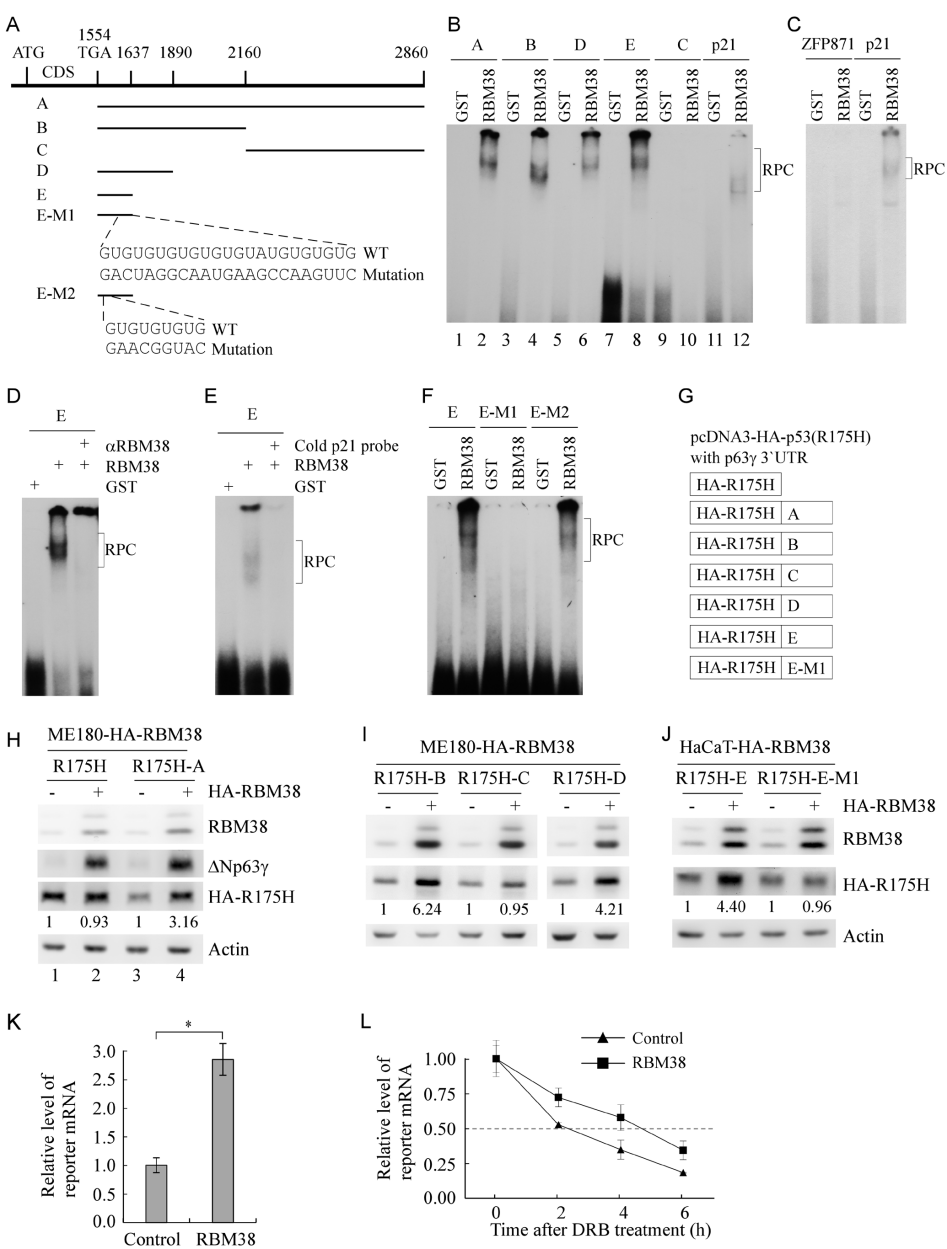
A GU-rich element in p63γ 3’UTR is bound by and responsive to RBM38 **A.** Schematic presentation of p63γ 3’UTR, probes used for REMSA assay, and mutation in two GU-rich elements in p63γ 3’UTR. **B.** RBM38 binds to probe A (nt 1554-2860), B (nt 1554-2160), D (nt 1554-1890), and E (nt 1554-1637), but not C (nt 2161-2860). REMSA assay was performed by mixing ^32^P-labeled RNA probes with recombinant GST or GST-fused HA-RBM38. The binding of RBM38 to p21 3’UTR was used as a positive control. The bracket indicates RNA-protein complexes (RPC). **C.** The binding assay of RBM38 to 3’UTR of ZFP871, a non-target of RBM38, was performed as a negative control. **D.** For super-shift assay, 3 µg of anti-HA antibody, which recognizes HA-tagged RBM38, was added to a reaction mixture containing probe E and GST-HA-RBM38. REMSA assay was performed as in *B*. **E.** For competition assay, an excess amount of unlabeled p21 cold probe was added to a reaction mixture containing RBM38 and probe E. **F.** RBM38 binds to probe E and E-M2, but not E-M1. E-M1 contains a mutation in the second GU-rich element whereas E-M2 contains a mutation in the first GU-rich element. **G.** Schematic presentation of reporters in which a fragment derived from p63γ 3’UTR was fused downstream of a mutant p53R(175H) reporter in pcDNA3-HA-p53R(175H). **H.** RBM38 increases HA-p53(R175H) expression from a reporter that carries the HA-p53(R175H) coding region plus fragment A from p63γ 3’UTR, but not from a reporter that carries only the HA-p53(R175H) coding region. ME180-HA-RBM38 cells, which were transfected with a reporter for 24 h, were split and then uninduced (-) or induced (+) to express RBM38 for 24 h. The levels of HA-RBM38, HA-p53(R175H), ΔNp63γ, and actin were determined by western blot analysis. The basal level of HA-p53(R175H) in RBM38-uninduced cells was arbitrarily set at 1.0 and the fold change was shown below each lane. **I.** RBM38 increases HA-p53(R175H) expression from reporters carrying fragments B and D, but not fragment C. The experiment was performed as in *H*. **J.** RBM38 increases HA-p53(R175H) expression from reporters carrying fragment E but fragment E-M1. The experiment was performed as in *H*. **K.** The mRNA level of a reporter carrying fragment E is increased by ectopic expression of RBM38. qRT-PCR was performed with total RNAs isolated from ME180-HA-RBM38 cells, which were transfected with a reporter for 24 h, split, and then uninduced (-) or induced (+) to express RBM38 for 12 h. The relative level of the reporter mRNA was normalized by the level of actin mRNA from three independent experiments. Asterisk indicates a significant difference (*p* = 0.00047). **L.** The mRNA half-life of the reporter carrying fragment E is prolonged by ectopic expression of RBM38. ME180-HA-RBM38 cells were transfected and induced as in *K*, and then followed by treatment with 20 μg/ml of 5,6-dichlorobenzimidazole 1-β-D-ribofuranoside (DRB), an inhibitor of transcription, for 0, 2, 4, and 6 h. The relative half-life of the reporter mRNA was calculated from triplicate samples and presented as Mean ± S.D.

The unique p63γ 3’UTR is 1,317-nt long (Figure 3A). To identify a potential binding region of RBM38 in p63γ 3’UTR in vitro, RNA electrophoretic mobility shift assay (REMSA) was performed by mixing recombinant GST or GST-fused HA-RBM38 with ^32^P-labeled probes A-E, derived from p63γ 3’UTR (Figure 3A). We found that RBM38 fusion protein directly bound to probes A, B, D, E, but not C (Figure 3B, lanes 1-10). The p21 3’UTR, known to contain an RBM38-binding site, was used as positive control (Figure 3B, lanes 11-12) [19, 22]. The ZFP871 3’UTR [28] without an RBM38-binding site was used as negative control (Figure 3C). Furthermore, we found that the probe E-protein complex was super-shifted with anti-RBM38 (Figure 3D) or inhibited by an excess amount of cold p21 probe (Figure 3E). These data suggest that the region (nt 1554-1637) is required for RBM38 to bind to p63γ 3’UTR. When searching this region, we found two GU-rich elements (Figure 3A). To test whether these GU-rich regions are response elements of RBM38, probe E-M1 with mutation in the second GU-rich region and probe E-M2 with mutation in the first GU-rich region were made (Figure 3A). We found that probe E-M1 but not E-M2 was unable to associate with RBM38 (Figure 3F).

To test whether p63γ 3’UTR upregulates the expression of a reporter gene in the presence of RBM38, we generated several reporters in which p63γ 3’UTRs were fused downstream of HA-tagged p53(R175H) (Figure 3G). We showed that ectopic expression of RBM38 induced the expression of both endogenous ΔNp63γ and exogenous HA-p53(R175H) from the reporter carrying the whole p63γ 3’UTR (A) in ME180 (Figure 3H, compare lanes 3 and 4) and HaCaT cells (Supplementary Figure 3, compare lanes 3 and 4). However, ectopic expression of RBM38 only increased the expression of endogenous ΔNp63γ but not exogenous HA-p53(R175H) from a control vector without a p63γ 3’UTR in ME180 and HaCaT cells (Figure 3H and Supplementary Figure 3, compare lanes 1 and 2). We would like to note that HA-p53(R175H) expression was inhibited by p63γ 3’UTR in the absence of RBM38 (Figure 3H and Supplementary Figure 3, compare lanes 1 and 3). Furthermore, we found that RBM38 increases HA-p53(R175H) expression from reporters carrying fragment B, D, or E, but not C or E-M1 in ME180 or HaCaT cells (Figure 3I*-3*J).

To determine whether RBM38 upregulates the reporter at the mRNA level, qRT-PCR analysis was performed and showed that ectopic expression of RBM38 significantly increased the mRNA level of the reporter carrying fragment E in ME180 cells (Figure 3K). To further explore whether RBM38 stabilizes the mRNA transcribed from a reporter plasmid, ME180-HA-RBM38 cells were transfected with the reporter carrying fragment E, uninduced or induced to express RBM38, and then treated with 5,6-Dichlorobenzimidazole 1-β-D-ribofuranoside (DRB) to inhibit nascent RNA synthesis. qRT-PCR analysis showed that the relative half-life of the reporter mRNA was increased from around 2.33 h in control cells to 4.68 h in RBM38-expressing cells (Figure 3L). Together, these data suggest that the second GU-rich element located within nt 1554-1637 of p63γ 3’UTR is required for RBM38 to enhance p63γ mRNA stability.

### Knockout of ΔNp63γ inhibits cell proliferation and migration in cells which primarily express ΔNp63 isoform

**Figure 4:**
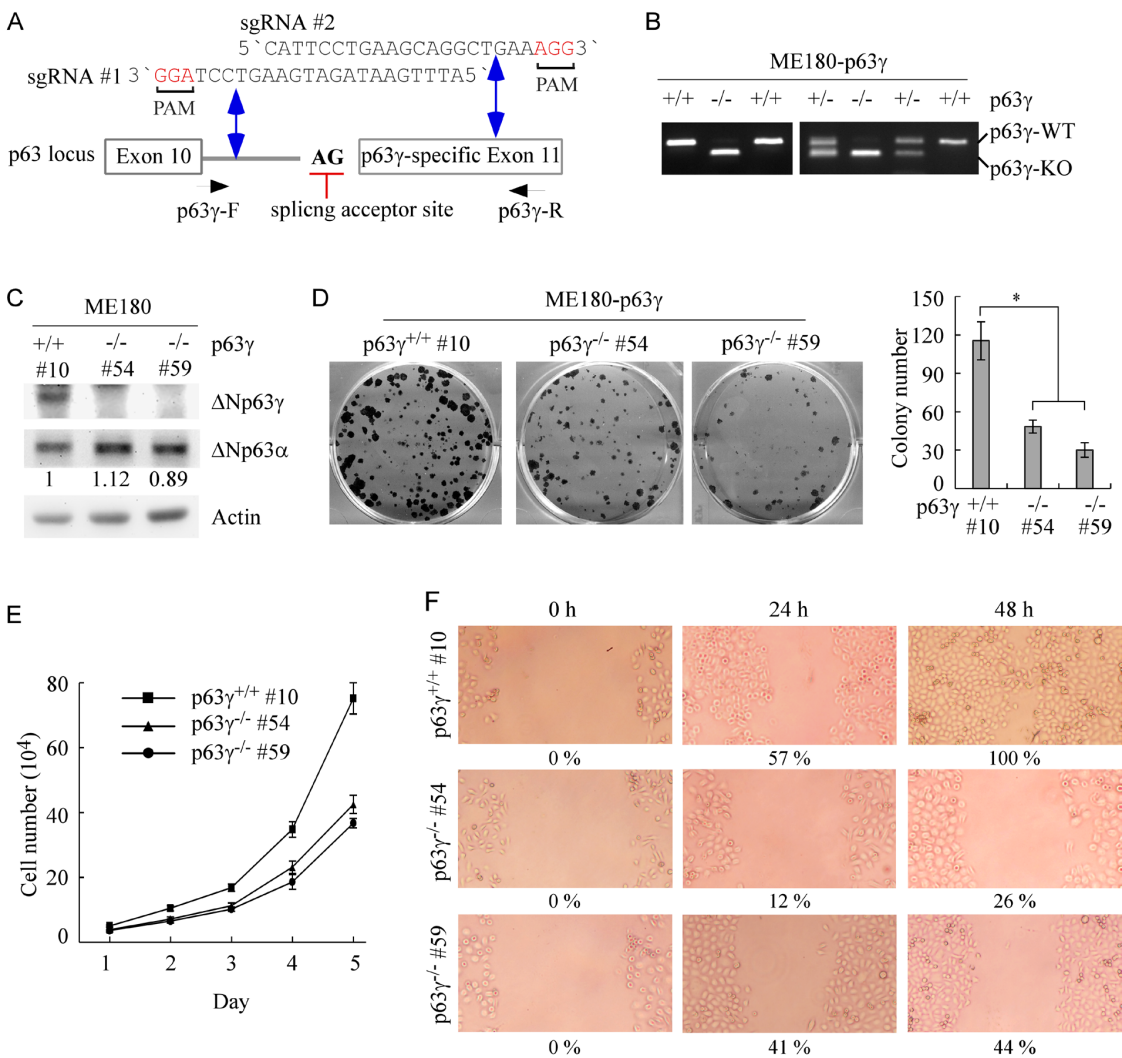
Knockout of ΔNp63γ inhibits cell proliferation and migration in ME180 cells, which primarily express ΔNp63 **A.** Schematic presentation of the locus of the *p63γ* gene, splice acceptor site (AG), and the p63γ-KO strategy with sgRNA #1-2 as indicated in intron 10 and exon 11. The predicted double strand breaks near the PAM motifs (in red) are indicated by double arrow. **B.** Genotyping of wildtype (WT), p63γ^+/-^, and p63γ^-/-^ ME180 cell lines. **C.** The level of ΔNp63γ protein is undetectable in p63γ^-/-^ ME180 cell lines. Western blots were performed with extracts from WT and p63γ^-/-^ ME180 cells, and then probed with antibodies against p63 and actin, respectively. **D.** *Left,* Knockout of *ΔNp63γ* inhibits colony formation. WT and p63γ^-/-^ ME180 cells were cultured for a period of 17 days, and then fixed and stained. *Right,* Quantification of the number of colonies with a diameter of >0.5 mm from three separate experiments. Asterisk indicates a significant difference (p63γ^-/-^ #54, *p* = 0.0262; p63γ^-/-^ #59, *p* = 0.0168). **E.** The number of WT and p63γ^-/-^ ME180 cells over a 5-d period was counted and presented as mean ± SD from three separate experiments. **F.** Wound healing assay was performed with WT and p63γ^-/-^ ME180 cells for a period of 48 h. The width of wound at each time point was measured and the ratio of wound healing at 0 h was arbitrarily set at 0 %. The ratio of wound healing at 24-48 h was calculated based on the ratio of wound width at 24-48 h with that in 0 h and shown below each lane.

TAp63 isoforms can transactivate genes involved in cell cycle arrest and apoptosis, suggesting that TAp63 isoforms are a tumor suppressor [2, 29]. Consistently, *TAp63*^*-/-*^ mice are prone to spontaneous tumors [16, 30]. By contrast, ΔNp63 isoforms are capable of promoting cell growth, suggesting that ΔNp63 isoforms have an oncogenic potential [2, 6]. However, the role of each individual isoform of TA and ΔNp63, especially the γ isoform, in cancer development and metastasis are still far from being fully understood. Overexpression of ΔNp63 frequently coincides with decreased expression of TAp63 in cells and tumors, and *vice versa* [7, 8, 24, 27]. Thus, ME180 and HaCaT cells, in which ΔNp63 isoforms are predominant whereas TAp63 isoforms are undetectable [24, 27], were used to examine the function of ΔNp63γ. For this purpose, we used CRISPR-Cas9 technology to generate p63γ knockout (KO) cell lines with two single guide RNAs (sgRNA), which target intron 10 and p63γ-specific exon 11, respectively (Figure 4A). The deletion of the splicing site of p63γ exon 11 was confirmed by PCR with a pair of primers as shown in Figure 4A. Several representative p63γ-KO clones of ME180 and HaCaT cells were shown in Figure 4B and Supplementary Figure 4A, respectively. DNA sequencing showed that a fragment of 51 bp (42 bp in intron 10 and 9 bp in exon 11: 5’-CCT AGG CCT TCA TTT TTT CTT TTC TCT GGT TCC TCT CTG CAG TCT CCT TTC-3’) was deleted from the *p63γ* gene. We found that in p63γ-KO ME180 and HaCaT cells, the level of ΔNp63γ protein was not detectable (Figure 4*C* and Supplementary Figure 4B). In addition, we showed that p63γ-KO had little, if any, effect on the level of ΔNp63α protein in ME180 (Figure 4C) and HaCaT cells (Supplementary Figure 4B). As ΔNp63γ has been implicated in transactivation of unique target genes [13], we tested whether p63γ-KO is capable of modulating ME180 and HaCaT cell growth. We found that in colony formation assays, p63γ-KO significantly inhibited the number and size of colonies in ME180 (Figure 4D) and HaCaT cells (Supplementary Figure 4C). Consistently, we found that p63γ-KO inhibited cell proliferation in ME180 cells over a 5-d period (Figure 4E) and prolonged cell doubling time from 1.02 d in WT cells to 1.22 d (clone #54) and 1.29 d (clone #59) in p63γ-KO cells.

It is known that p63 regulates adhesion and migration of epithelial cells [17, 31]. Thus, wound healing assay was performed to measure the effect of p63γ-KO on cell migration in ME180 and HaCaT cells. We found that upon p63γ-KO, the migration of ME180 (Figure 4F) and HaCaT (Supplementary Figure 4D) cells was inhibited during a 48-h period. Together, these findings suggest that ΔNp63γ has an ability to promote cell proliferation and migration.

### Knockout of TAp63γ promotes cell proliferation and migration in MIA PaCa-2 cells which primarily express TAp63 isoform

**Figure 5:**
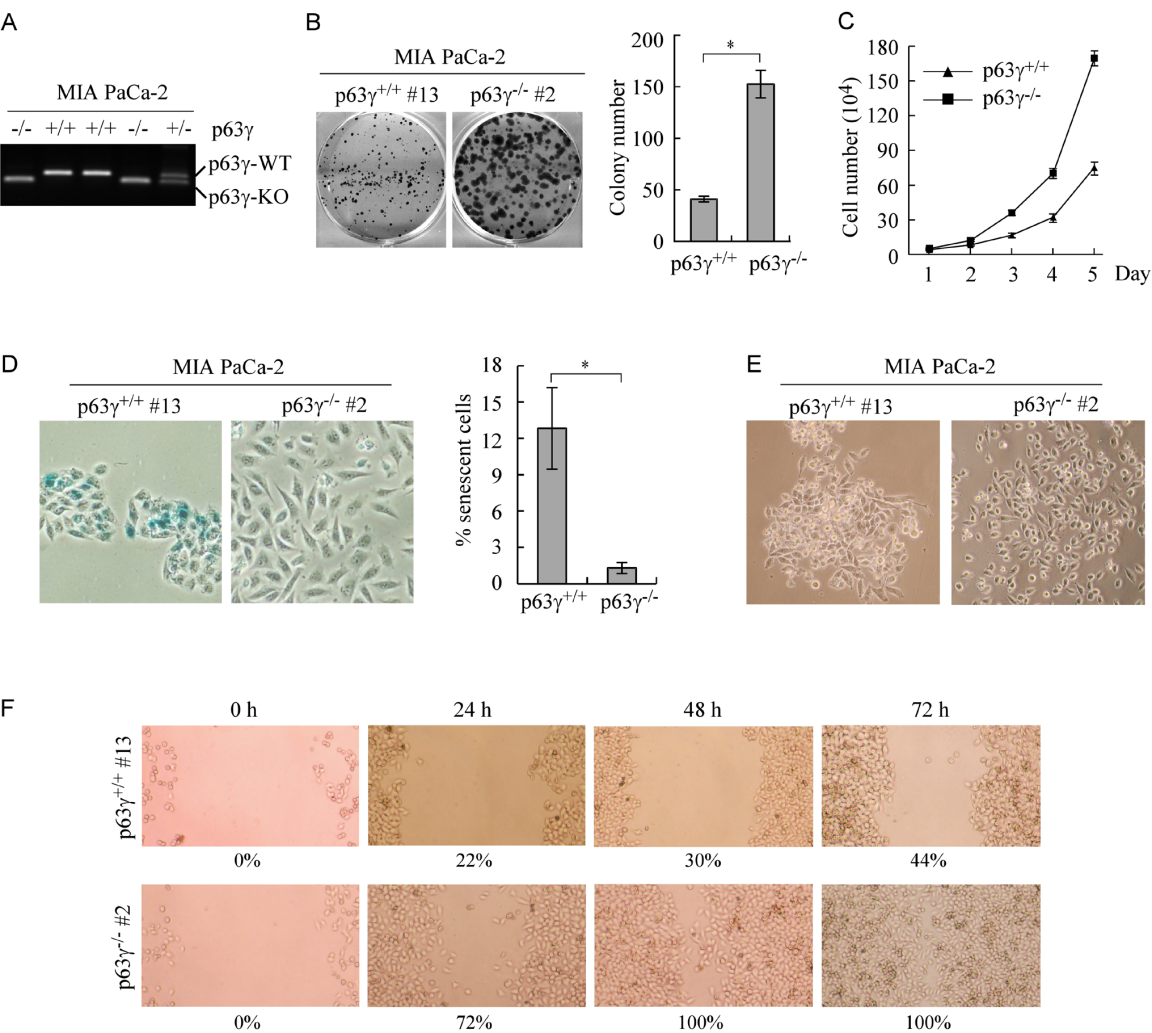
Knockout of TAp63γ promotes cell proliferation and migration in MIA PaCa-2 cells, which primarily express TAp63 **A.** Genotyping of WT, p63γ^+/-^, and p63γ^-/-^ MIA PaCa-2 cell lines. **B.** *Left,* Knockout of *p63γ* promotes colony formation. WT and p63γ^-/-^ MIA PaCa-2 cells were cultured for a period of 13 days, and then fixed and stained. *Right,* Quantification of the number of colonies with a diameter of >0.5 mm from three separate experiments. Asterisk indicates a significant difference (*p* = 0.0075). **C.** The number of WT and p63γ^-/-^ MIA PaCa-2 cells over a 5-d period was counted and presented as mean ± SD from three separate experiments. **D.** *Right,* Representative images of SA-β-Gal-stained wild-type and p63γ^-/-^ MIA PaCa-2 cells. *Left,* The percentage of SA-β-Gal-positive cells shown as in *Right*. Asterisk indicates a significant difference (*p* = 2.1E-05). **E.** Knockout of *p63γ* changes the pattern of cell growth. Representative image of single clone of WT or p63γ^-/-^ MIA PaCa-2 cells captured with a phase-contrast microscopy. *F*, Wound healing assay was performed with WT or p63γ^-/-^ MIA PaCa-2 cells for a period of 72 h. The ratio of wound healing was calculated as in *Figure **4F*.

To test whether TAp63γ regulates cell proliferation and migration, we generated multiple p63γ-KO MIA PaCa-2 cell lines (Figure 5A) with the same strategy as shown in Figure 4A. MIA PaCa-2 cell line primarily expresses TAp63 but undetectable ΔNp63 [27]. Colony formation assay and growth curve assay were performed and showed that p63γ-KO MIA PaCa-2 cells were highly competent in cell proliferation as compared to isotype control cells (Figure 5B*-5*C). Knockout of p63γ decreased cell doubling time from 0.99 d in WT MIA PaCa-2 cells to 0.78 d in p63γ-KO cells. We also found that the number of SA-β-galactosidase (SA-β-gal)-positive cells was significantly decreased in p63γ-KO MIA PaCa-2 cells as compared to that in control cells (Figure 5D). In addition, we found that p63γ-KO changed the pattern of cell growth and made MIA PaCa-2 cells spread out (Figure 5E). Consistently, wound healing assay showed that p63γ-KO promoted the ability of MIA PaCa-2 cells to migrate during a 72-h period (Figure 5F). Together, these findings suggest that TAp63γ inhibits cell proliferation and migration.

## DISCUSSION

The *p63* gene encodes for TA and ΔN isoforms. In addition, both TAp63 and ΔΝp63 contain five C-terminal splicing isoforms (α-ε) [1]. Due to their differences in C-terminal sterile alpha motif and inhibitory domain, these p63 isoforms differentially regulate their target genes, which are responsible for their strong or weak activity in apoptosis, cell cycle arrest, and senescence [1, 16, 29, 32]. Thus, how each p63 individual isoform is regulated may determine its distinct function and is an area of intense investigation. Previously, we found that the expression of p63α and β isoforms is inhibited by RBM38 and RBM24, both of which bind to the AU-/U-rich elements in 3’ UTRs of p63α and β transcripts [24, 33]. In this study, we found that the levels of ΔNp63γ transcript and protein are highly induced upon ectopic expression of RBM38. Importantly, RBM38 knockdown results in a reduction of endogenous ΔNp63γ transcript and protein. Conversely, the levels of ΔNp63β transcript and protein are decreased by ectopic expression of RBM38 but increased by RBM38 knockdown, consistent with our previous report [24]. Interestingly, we found that the half-life of p63γ mRNA is prolonged by RBM38 via a GU-rich element in p63γ 3’UTR. The GU-rich element is also responsive to RBM38 as evidenced by increased expression of a reporter that contains p63γ 3’UTR with an intact GU-rich element. These data suggest that RBM38 is capable of stabilizing p63γ transcript but destabilizing p63α and β transcripts most likely due to the presence of entirely different RBM38-responsive elements: a GU-rich element in p63γ 3’UTR but AU-/U-rich elements in p63α and β 3’UTRs [24].

It is well known that different p63 isoforms, especially TAp63γ and ΔNp63γ, display common and unique transcriptional activities in gene regulation. For example, both TAp63γ and ΔNp63γ, but not TAp63α and ΔNp63α, can induce expression of GPX2, which decreases the sensitivity of cells to oxidative stress-induced cell death [13]. In addition, TAp63γ, but not ΔNp63γ, has a strong activity in inducing expression of Glyoxalase II [34], p21 [3], IGFBP3 [3, 35], and PIG3 [3]. Since TAp63γ and ΔNp63γ exhibit such a distinct activity in gene regulation, we postulated that TAp63γ and ΔNp63γ may play a unique role in cancer development and progression. Indeed, we found that cell proliferation and cell migration are inhibited by knockout of ΔNp63γ in ME180 and HaCaT cells. In contrast, knockout of TAp63γ in MIA PaCa-2 cells promotes cell proliferation and migration and inhibits cellular senescence. In conclusion, these findings suggest that ΔNp63γ has an oncogenic potential whereas TAp63γ is a tumor suppressor. Thus, modulation of ΔNp63γ and TAp63γ expression via RNA-binding proteins may be explored for a new therapeutic approach to manage tumors with overexpressed ΔNp63γ or diminished TAp63γ.

## MATERIALS AND METHODS

### Antibodies and western blot analysis

Anti-RBM38 was purified from rabbit sera against His-tagged RBM38 protein through GST-RBM38 beads. Other antibodies used for western blot assays included anti-actin (Sigma), anti-p63 (4A4, Santa Cruz), and anti-HA (Covance). Immunoblots were visualized by SuperSignal West Femto Chemiluminiscent detection reagents (Pierce).

### Plasmids and small interference RNA oligos

To knock out the human *p63γ* gene, two single-guide RNA (sgRNA) expression vectors pSpCas9(BB)-2A-Puro-sgp63γ-1 and pSpCas9(BB)-2A-Puro-sgp63γ-2 were used to target intron 10 and exon 11 of the *p63γ* gene, respectively, and delete a fragment of 51 bp (42 bp in intron 10 and 9 bp in exon 11) in the splicing sit of exon 11. The generation of sgRNA expression vectors were performed as described previously [36]. The oligonucleotides for sgp63γ-1 are sense, 5’-CAC CGA TTT GAA TAG ATG AAG TCC T-3’, and antisense, 5’-AAA CAG GAC TTC ATC TAT TCA AAT C-3’. The oligonucleotides for sgp63γ-2 are sense, 5’-CAC CGC ATT CCT GAA GCA GGC TGA A-3’, and antisense, 5’-AAA CTT CAG CCT GCT TCA GGA ATG C-3’.

The expressing reporters were generated by cloning p63γ 3’UTR into pcDNA3-HA-p53(R175H) at the direct downstream of p53(R175H). Whole p63γ 3’UTR (fragment A: nt 1554-2860) was amplified with forward primer P1, 5’-CTC GAG AGC CCT ATC TCT ATA TTT TAA GTG-3’, and reverse primer P2, 5’-TCT AGA AAA CCA AGA TGC AAA AGT TTA TTG-3’. p63γ 3’UTRs with partial deletions were amplified with forward primer P1 and reverse primer, 5’-TCT AGA ACT CTC CAC CCA CAA AGT TG-3’ (fragment B: nt 1554-2160), 5’-TCT AGA TTC CAA TAA ACA TCT TAA TG-3’ (fragment D: nt 1554-1890), or 5’-CTC GAG TAT CTA GCC CTC ATA AAC AG-3’ (fragment E: nt 1554-1637). Fragment C (nt 2161-2860) was amplified with forward primer, 5’-CTC GAG TCT TTG TGA GAA CTT GCA TTA TTT G-3’, and reverse primer P2. Fragment E-M1, which carries mutation in the second GU-rich region, was amplified with forward primer P1 and reverse primer, 5’-TCT AGA CAC ACG GAA CTT GGC TTC ATT GCC TAG TCT CAC ATA TAC ACA TGG AAA TAC-3’. The resulting fragments were subcloned into pGEM-T easy vector. Upon confirmation by sequencing, the fragments were fused downstream of p53(R175H) in pcDNA3-HA-p53(R175H).

All small interfering RNAs (siRNAs) were purchased from Dharmacon RNA Technologies (Chicago, IL). To transiently knock down human endogenous p63γ or RBM38, one siRNA against p63γ, 5’-GCC ACU AGU GAG AGA AUC U dTdT-3’, or one siRNA against RBM38, 5’-ACC UUG AUC CAG CGG ACU U dTdT-3’, was transfected into the cells at the final concentration of 30 nM for 1-3 days. The scrambled siRNA, 5’-GCA GUG UCU CCA CGU ACU A dTdT-3’, was used as a control.

### Cell lines

All cell lines were cultured in DMEM (Invitrogen) medium supplemented with 10% fetal bovine serum (Hyclone). ME180 and HaCaT cell lines, which inducibly express HA-tagged RBM38, were described previously [24]. To generate MIA PaCa-2, HaCaT, and ME180 cell lines with p63γ-KO, two sgRNA expression vectors pSpCas9(BB)-2A-Puro-sgp63γ-1 and pSpCas9(BB)-2A-Puro-sgp63γ-2 were cotransfected into cells. The resulting p63γ-KO cell lines were selected with puromycin and further confirmed by PCR amplification of the *p63γ* gene with forward primer 5’-GAG TGT GTT TCT GAA TTC AAT TG-3’ and reverse primer 5’-AGG GCT CTA TGG GTA CAC TG-3’. The p63γ-KO cell lines and isotype control cell lines were further confirmed by Western blot analysis with anti-p63 antibody.

### RNA purification, RT-PCR, and quantitative RT-PCR

Total RNA was isolated from cells using TRIzol reagent (Invitrogen). cDNA was synthesized using Iscript™ cDNA synthesis kit (Bio-Rad). To quantify the levels of p63γ mRNA, regular RT-PCR or quantitative RT-PCR (qRT-PCR) was performed with forward primer 5’-ACA CAC ATG GTA TCC AGA TG-3` and reverse primer 5`-TTC CTG AAG CAG GCT GAA AG-3’. The level of p63β mRNA was measured with forward primer 5’-CAT GAA CAA GCT GCC TTC TG-3’ and reverse primer 5’-CTT GCC AGA TCC TGA CAA TG-3’. The level of RBM38 was measured with forward primer 5’-CTG AGA GGG CTT GCA AAG AC-3’ and reverse primer 5’-CAC GAT GGC TGG TGG GTA G-3’. Actin mRNA was measured as an internal control with forward primer 5’-TCC ATC ATG AAG TGT GAC GT-3’ and reverse primer 5’-TGA TCC ACA TCT GCT GGA AG-3’.

### RNA-protein immunoprecipitation assay

RNA-protein immunoprecipitation assay was carried out as previously described [37]. Briefly, ME180-HA-RBM38 cells were uninduced or induced to express RBM38 for 24 h and then lysed at 4°C with a lysis buffer (50 mM Tris-HCl, pH 7.4, 1% NP-40, 150 mM NaCl, 1× PIC, 1 mM PMSF, 0.5 U/μl RNasin). Five percent of the cell extracts were used for total RNA isolation and the remaining extracts were incubated with protein A/G beads conjugated with anti-HA antibody or a control IgG overnight at 4°C. Following four washes with a buffer containing RNase-free DNase, RNAs on the beads were purified with TRIzol reagent. RT-PCR was performed to detect the levels of p63γ mRNA bound by RBM38 as described above. The actin mRNA was amplified as a negative control.

### RNA electrophoretic mobility shift assay (REMSA)

p63γ 3’UTRs were PCR-amplified using forward primers containing T7 promoter sequence and reverse primers as listed in Table 1. The p21 [19] and ZFP871 [28] probes were generated as previously described and used as positive and negative control, respectively. RNA probes were made from *in vitro* transcription with T7 RNA polymerase in the presence of α-^32^P-UTP. GST-tagged RBM38 protein was purified as described previously [38]. REMSA was performed with 200 nM recombinant protein, 1 mg/ml of yeast tRNA, and 50,000 CPM ^32^P-labeled RNA probe in a reaction buffer (10 mM HEPES, pH 8.0, 10 mM KCl, 10 mM MgCl_2_, 1 mM DTT) for 20 min at 25°C. RNA/protein complexes were digested with 100 U RNaseT1 for 15 min at 37°C and then separated in 6% of native PAGE. RNA-protein complexes were visualized by autoradiography. To test the specificity of RBM38 binding to p63γ 3’UTR, competition assay was performed by adding an excess amount of unlabeled p21 3’UTR cold probe into the reaction mixture with a ^32^P-labeled probe as described previously [19].

**Table 1:** Primers used to amplify fragments A-E of p63γ 3`UTR

Probe templetes	Primers	Sequences
A (nt 1554-2860)	Forward	5`-GGATCCTAATACGACTCACTATAGGGAGAGCCCTATCTCTATATTTTAA-3`
	Reverse	5`-AAACCAAGATGCAAAAGTTTATTG-3`
B (nt 1554-2160)	Forward	5`-GGATCCTAATACGACTCACTATAGGGAGAGCCCTATCTCTATATTTTAA-3`
	Reverse	5`-ACTCTCCACCCACAAAGTTG -3`
C (nt 1554-1890)	Forward	5`-GGATCCTAATACGACTCACTATAGGGAGAGCCCTATCTCTATATTTTAA-3`
	Reverse	5`-TTCCAATAAACATCTTAATG-3`
D (nt 1554-1637)	Forward	5`-GGATCCTAATACGACTCACTATAGGGAGAGCCCTATCTCTATATTTTAA-3`
	Reverse	5`-ACACACGCACACACATACAC-3`
E (nt 2160-2860)	Forward	5`- GGATCCTAATACGACTCACTATAGGGAGTCTTTGTGAGAACTTGCATT-3`
	Reverse	5`-AAACCAAGATGCAAAAGTTTATTG-3`
D-M1	Forward	5`-GGATCCTAATACGACTCACTATAGGGAGAGCCCTATCTCTATATTTTAA-3`
	Reverse	5`-ACACACGGAACTTGGCTTCATTGCCTAGTCTCACATATACACATGGAAATAC-3`
D-M2	Forward	5`-AGCCCTATCTCTATATTTTAAGAACGGTACTTGTATTTCCATGTGTATATG-3`
	Reverse	5`-ACACACGCACACACATACAC-3`

### mRNA half-life assay

To measure the stability of p63γ mRNA, ME180-HA-RBM38 cells were uninduced or induced to express RBM38 for 12h, and then treated with 5 μg/ml of actinomycin D (Act D), an inhibitor of transcription, for 0, 2, 4, 6, 8 or 10 h. The relative level of p63γ mRNA were quantified by qRT-PCR and normalized by the level of actin mRNA from three separate experiments, which were then plotted versus time to calculate the half-life of p63γ mRNA.

To measure reporter mRNA stability, ME180-HA-RBM38 cells were transfected with a reporter plasmid pcDNA3-HA-p53(R175H)-E for 24 h and then split into 3.5 cm dishes. The cells were uninduced or induced to express RBM38 for 12h, and then treated with 20 μg/ml of 5,6-dichlorobenzimidazole 1-β-D-ribofuranoside (DRB), an inhibitor of transcription, for 0, 2, 4, and 6 h. The relative levels of the reporter mRNA were quantified by qRT-PCR with forward primer 5’-GCT GAA TGA GGC CTT GGA AC-3’ and reverse primer 5’-ACA TAT ACA CAT GGA AAT ACA AC-3’, normalized by the levels of actin mRNA from three separate experiments, and then plotted versus time to calculate the half-life of the reporter mRNA.

### Colony formation assay, growth curve, cellular senescence assays, and wound healing assay

For Colony formation assay, ME180, HaCaT, or MIA PaCa-2 cells (1000 per well) in six-well plates were cultured for 13 to 17 days. The clones were fixed with methanol/glacial acetic acid (7:1) and then stained with 0.1% of crystal violet.

For growth curve assay, cells were split and seeded at a density of 2.5×10^4^ cells per well in a 6-well plate. At the times indicated, cells were rinsed with PBS to remove dead cells. Live cells on the plate were trypsinized and counted using the Coulter cell counter (Beckman Coulter, Fullerton, CA). The average number of cells from three wells was used for cell proliferation determination. The cell doubling time was calculated with formula: Doubling time = duration*log(2)/(log(final number)−log(initial number)).

Cellular senescence assay was performed as previously described [39]. The percentage of senescent cells was calculated based on the number of positive cells among 2,000 cells.

For wound healing assay, the cells were seeded at a density of 45×10^4^ cells per well in a 6-well plate cells and grown for 24 h. The monolayers were wounded by scraping with a P200 micropipette tip and washed two times with PBS. At indicated time points after scraping, cell monolayers were photographed with phase contrast microscopy. Cell migration was determined by visual assessment of cells migrating into the wound.

### Statistical analysis

Two-group comparisons were analyzed by two-sided Student’s *t* test. *p* values were calculated and *p* < 0.05 was considered significant.

## SUPPLEMENTARY MATERIALS FIGURES


