# Supplementary material for: TAp63γ and ΔNp63γ are regulated by RBM38 via mRNA stability and have an opposing function in growth suppression
Source: Oncotarget. 2017 Jun 13;8(45):78327–39. doi: 10.18632/oncotarget.18463 (PMC5667965; doi:10.18632/oncotarget.18463)
Supplement: Supplementary file 1 [file oncotarget-08-78327-s001.pdf]

TAp63γ and ΔNp63γ are regulated by RBM38 via mRNA stability and have an opposing function in growth suppression

SUPPLEMENTARY MATERIALS

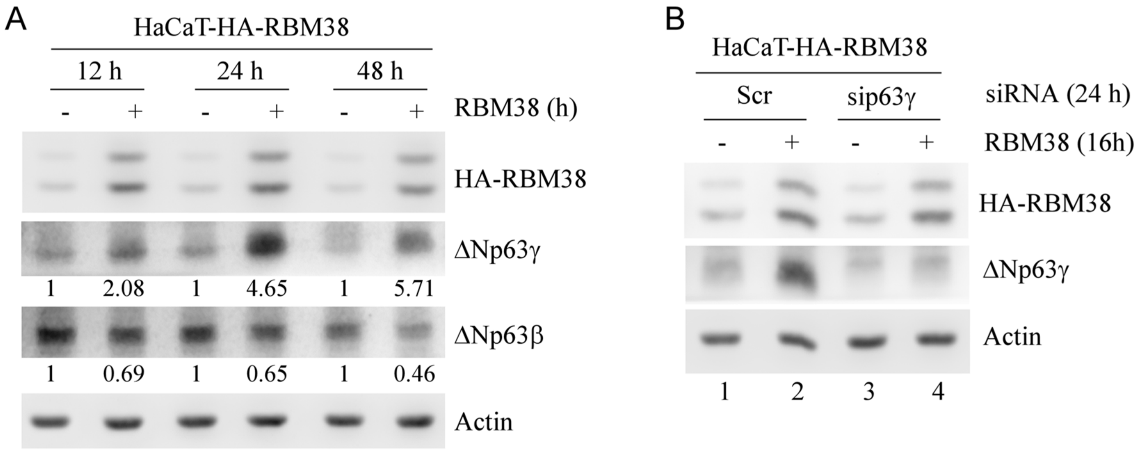

Supplementary Figure 1: The levels of ΔNp63γ and ΔNp63β proteins are differentially regulated by ectopically expressed RBM38 in HaCaT cells. A, Ectopic expression of RBM38 increases the level of ΔNp63γ protein but decreases the level of ΔNp63β protein. Western blots were prepared with extracts from HaCaT-HA-RBM38 cells, which were uninduced (-) or induced (+) to express RBM38 for 12, 24, or 48 h, and then probed with antibodies against p63, HA, and actin, respectively. The basal level of ΔNp63γ or ΔNp63β in cells without RBM38 expression was arbitrarily set at 1.0 and the relative fold change was shown below each lane. B, A p63γ siRNA decreases the level of basal and RBM38-induced ΔNp63γ protein. Western blots were prepared with extracts from HaCaT-HA-RBM38 cells, which were transfected with scrambled siRNA or siRNA again p63γ for 8 h and then uninduced (-) or induced (+) to express RBM38 for 16 h, and then probed as in *A*.

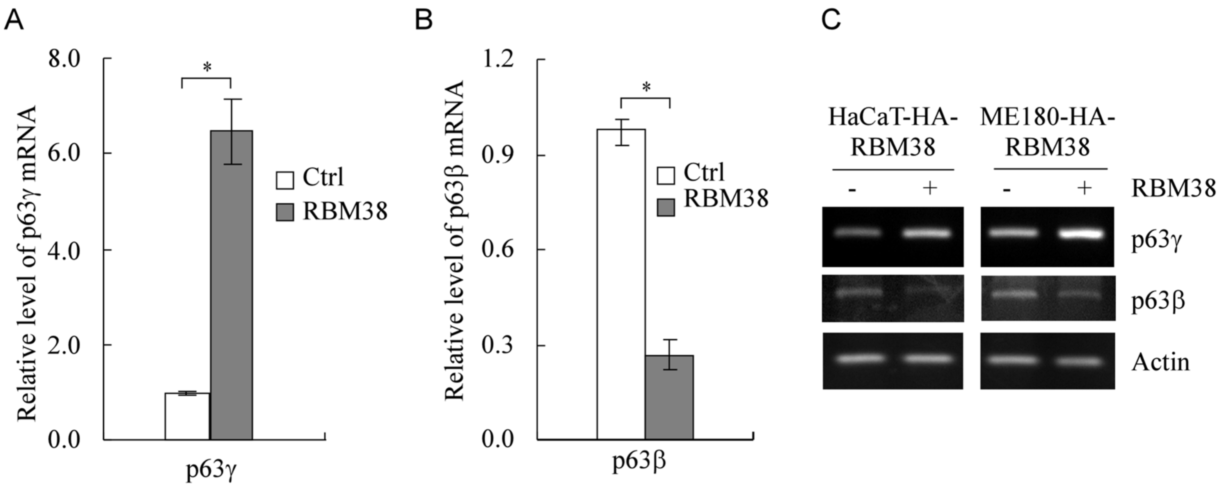

Supplementary Figure 2: Ectopically expressed RBM38 increases the level of p63γ transcripts. A, The level of p63γ transcripts is increased by ectopic expression of RBM38. Quantitative RT-PCR (qRT-PCR) was performed with total RNAs isolated from HaCaT-HA-RBM38 cells, which were uninduced (-) or induced (+) to express RBM38 for 24 h. The relative level of p63γ mRNA was normalized by the level of actin mRNA from three independent experiments. Asterisk indicates a significant difference (p=0.00014). B, The level of p63β transcripts is decreased by ectopic expression of RBM38. The experiment was performed the same as in (*A*) except for that p63β primers were used. Asterisk indicates a significant difference (p=0.0000227). C, The levels of p63γ, p63β, and actin transcripts were measure by regular RT-PCR with total RNAs isolated from HaCaT-HA-RBM38 or ME180-HA-RBM38 cells, which were uninduced (-) or induced (+) to express RBM38 for 24 h.

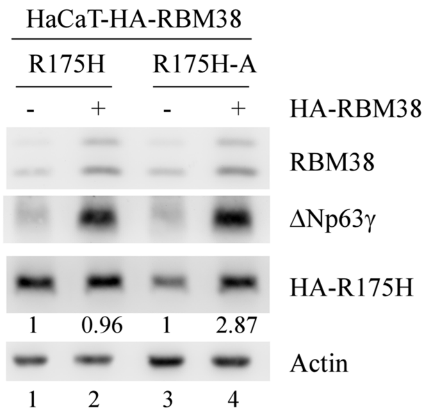

Supplementary Figure 3: The expression reporter carrying fragment A derived from p63γ 3'UTR is responsive to RBM38 in HaCaT cells. HaCaT-HA-RBM38 cells were transfected with pcDNA3-HA-p53(R175H) or pcDNA3-HA-p53(R175H)-A expression vector for 24 h, followed with (+) or without (-)RBM38 expression for 24 h. Western blot assay was used to detect the expression of HA-RBM38, p63γ, HA-p53(R175H), and actin. The basal level of HA-p53(R175H) in control cells was arbitrarily set at 1.0 and the relative fold change was shown below each lane.

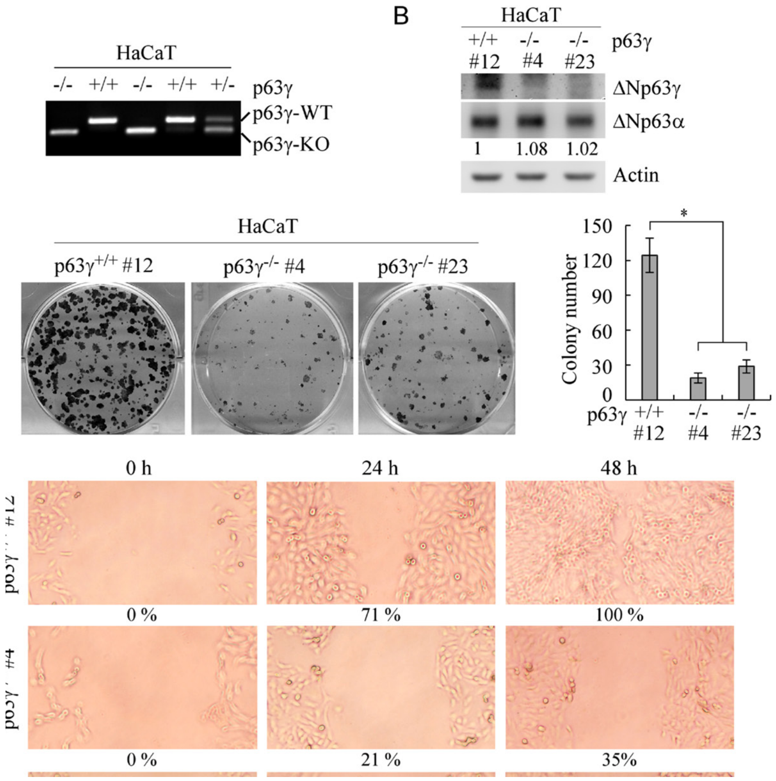

Supplementary Figure 4: Knockout of the p63γ gene inhibits cell proliferation and migration in ΔNp63-expressing HaCaT cells. A, Genotyping of the p63 gene in WT, p63γ$^{+/-}$, and p63γ$^{-/-}$ HaCaT cell lines. B, The level of ΔNp63γ protein is undetectable in p63γ$^{-/-}$ HaCaT cell lines. Western blots were performed with extracts from WT and p63γ$^{-/-}$ HaCaT cells, and then probed with antibodies against p63 and actin, respectively. C, Left, Knockout of the p63 gene inhibits cell proliferation in HaCaT cells. Colony formation assay was performed with WT and p63γ$^{-/-}$ HaCaT cells (1000 per well in six-well plates) for a period of 17 days. Right, Quantification of the number of colonies with a diameter of >0.5 mm from three separate experiments. Asterisk indicates a significant difference (p63γ$^{-/-}$ #4, p=0.0105; p63γ$^{-/-}$ #23, p=0.0136). D, Knockout of the *p63γ* gene inhibits cell migration in HaCaT cells. Wound healing assay was performed with WT and p63γ$^{-/-}$ HaCaT cells for a period of 48 h. The width of wound at each time point was measured and the ratio of wound healing at 0 h was arbitrarily set at 0 %. The ratio of wound healing at 24-48 h was calculated based on the ratio of wound width at 24-48 h with that in 0 h and shown below each lane.
